# Heterophylly Quantitative Trait Loci Respond to Salt Stress in the Desert Tree *Populus euphratica*

**DOI:** 10.3389/fpls.2021.692494

**Published:** 2021-07-15

**Authors:** Yaru Fu, Feiran Li, Shuaicheng Mu, Libo Jiang, Meixia Ye, Rongling Wu

**Affiliations:** ^1^Center for Computational Biology, College of Biological Sciences and Technology, Beijing Forestry University, Beijing, China; ^2^Departments of Public Health Sciences and Statistics, Center for Statistical Genetics, The Pennsylvania State University, Hershey, PA, United States

**Keywords:** heterophylly, leaf shape, salt stress, QTL, *Populus euphratica*

## Abstract

Heterophylly, or leaf morphological changes along plant shoot axes, is an important indicator of plant eco-adaptation to heterogeneous microenvironments. Despite extensive studies on the genetic control of leaf shape, the genetic architecture of heterophylly remains elusive. To identify genes related to heterophylly and their associations with plant saline tolerance, we conducted a leaf shape mapping experiment using leaves from a natural population of *Populus euphratica*. We included 106 genotypes grown under salt stress and salt-free (control) conditions using clonal seedling replicates. We developed a shape tracking method to monitor and analyze the leaf shape using principal component (PC) analysis. PC1 explained 42.18% of the shape variation, indicating that shape variation is mainly determined by the leaf length. Using leaf length along shoot axes as a dynamic trait, we implemented a functional mapping-assisted genome-wide association study (GWAS) for heterophylly. We identified 171 and 134 significant quantitative trait loci (QTLs) in control and stressed plants, respectively, which were annotated as candidate genes for stress resistance, auxin, shape, and disease resistance. Functions of the stress resistance genes *ABSCISIC ACIS-INSENSITIVE 5-like* (*ABI5*), *WRKY72*, and *MAPK3* were found to be related to many tolerance responses. The detection of *AUXIN RESPONSE FACTOR17-LIKE* (*ARF17*) suggests a balance between auxin-regulated leaf growth and stress resistance within the genome, which led to the development of heterophylly *via* evolution. Differentially expressed genes between control and stressed plants included several factors with similar functions affecting stress-mediated heterophylly, such as the stress-related genes *ABC transporter C family member 2* (*ABCC2*) and *ABC transporter F family member* (*ABCF*), and the stomata-regulating and reactive oxygen species (ROS) signaling gene *RESPIRATORY BURST OXIDASE HOMOLOG* (*RBOH*). A comparison of the genetic architecture of control and salt-stressed plants revealed a potential link between heterophylly and saline tolerance in *P. euphratica*, which will provide new avenues for research on saline resistance-related genetic mechanisms.

## Introduction

Heterophylly, or leaf morphological changes along plant shoot axes, is an important plant strategy for adapting to heterogeneous environments (Winn, 1999; Li et al., 2019). For example, juvenile *Bromeliaceae* plants have narrow leaves to conserve water, whereas adults have larger flat leaves that support an increased transpiration (Benzing, 2000). Unlike studies on phenotypic plasticity, which is a mechanism by which plants develop adaptive traits (Donohue, 2013), investigations of heterophylly allow us to better understand both leaf development and phase change regulation (Jones, 1999; Nakayama et al., 2012). To date, such studies have contributed greatly to elucidating the mechanisms involved in the ecology, evolution, and development of leaf functions.

The timing and position of leaf ontogeny are decisive factors in determining leaf morphology. Expression of the evolutionarily conserved SQUAMOSA-PROMOTER BINDING PROTEIN-LIKE (SPL) module miR156, and the opposing miR172 module, is crucial for plant transition from juvenile to adult (Wang, 2016). In *Populus* × *canadensis* trees, miR156 expression was greatest in leaves at the base of the shoot and decreased upward along the shoot, suggesting a conserved role in mediating tree heterophylly (Wang et al., 2011). miR164 overexpression has been found to inhibit the expression of *CUP-SHAPED COTYLEDON* (*CUC2/CUC3*), leading to leaf serration deficiency (Rubio-Somoza et al., 2014). In aquatic plants, endogenous abscisic acid (ABA) has been reported to control leaf morphology (Goliber and Feldman, 1989), and an ABA-responsive heterophylly (*ABRH*) gene was found to respond to early heterophylly induction (Hsu et al., 2001). Despite extensive efforts to identify heterophylly genes and related modules, few studies have linked the patterns in heterophylly variation to stress tolerance mechanisms, and the genetic architecture regulating stress tolerance-induced heterophylly has rarely been studied.

*Populus euphratica* is a dominant tree species in the deserts of China. *P. euphratica* has evolved high salt and drought tolerance and is used as a model species for stress tolerance research due to its important role in maintaining the ecological balance within its arid habitat (Ma et al., 2013). *P. euphratica* exhibits a broad range of heterophylly, including strip-shaped, lanceolate, ovate, and broad ovate leaf shapes, depending on the developmental stage of the tree. Strip and lanceolate leaves tend to disappear over time, leaving more ovate or broad ovate leaves as the tree matures. The diversity of *P. euphratica* leaf morphology has promoted its survival in harsh desert environments. A study of the microanatomy of four types of leaf demonstrated that broad ovate leaves had a stronger xeromorphic structure, characterized by differences in stoma shape and density and in the size of the lower stomatal chamber compared with strip, lanceolate, and ovate leaves (Liu et al., 2015); these differences suggest that the transition toward ovate leaves is associated with adaptation to drought and strong light conditions (Li and Zheng, 2005). The physiology of broad ovate leaves indicates higher efficiency of both photosynthesis and water use, as well as higher osmotic adjustment capacity, compared with the three other leaf shapes (Liu et al., 2015). Therefore, heterophylly provides both physiological and morphological advantages for *P. euphratica* in terms of surviving abiotic stress. Many studies of heterophylly have used physiological approaches and microdissection data to examine its mechanisms; however, these have all focused on changes in leaf shape within individual trees. In its natural distribution, *P. euphratica* must adapt to saline stress conditions from the seedling stage until maturity. The coordination between growth and stress tolerance, i.e., iterative trade-offs between defense and growth at the juvenile stage, may lead to optimized leaf growth (Smakowska et al., 2016), such that the modification of leaf shapes over time leads to heterophylly patterns. However, few studies have examined whether salt stress affects heterophylly patterns or the potential genetic architecture for leaf heterophylly regulation in the context of such salt stress responses.

Functional identification of heterophylly genes remains challenging due to the lack of a genetic transformation system and long tree life cycles. Genome-wide association study (GWAS) methods can be applied for highly accurate mapping of trait-associated loci, to elucidate the genetic architecture of heterophylly. In this process, heterophylly is quantitatively defined as a multivariate phenotype (e.g., in terms of length, width, and the length/width ratio along the shoot axis) or precisely measured using geometric morphometrics, e.g., multiple radius centroid contour (RCC) curves of outline coordinates along the leaf margin (Fu et al., 2018; Sun et al., 2018). Functional mapping techniques facilitate the study of genetic control over trait formation using dynamic models that integrate the pathways underlying phenotypic formation (Ma et al., 2002; Sun and Wu, 2015). Such models identify the genes involved in dynamic phenotype formation processes and quantify the patterns of genetic effects over time. This approach can be applied to map heterophylly genes by examining shape phenotypes along the shoot axis as a quasi-dynamic function of growth. Changes in heterophylly patterns can be estimated for allelic genotypes at quantitative trait loci (QTLs) to determine how specific genes control heterophylly to mediate saline stress responses.

In this study, we analyzed a natural population of *P. euphratica* along the Tarim River in Xinjiang, China. We used new shoots sprouted from juvenile branches as explants for tissue culture to produce seedling plants. Propagated seedlings for each genotype line were employed to derive control and saline-treated groups from the sample population. Because all propagated seedlings within the population were at the juvenile stage, heterophylly observations in this study focused only on lanceolate leaves along the shoot axis during the first growing season. We integrated the shape indicator data collected at different positions along the shoot axis to establish a multidimensional leaf phenotype and to express heterophylly quantitatively. In addition, single-nucleotide polymorphism (SNP) data were collected for all genotypes for GWAS and functional mapping. Heterophylly patterns of the control and saline-treated groups were then compared in terms of differentially expressed genes to determine whether heterophylly is involved in saline tolerance in *P. euphratica*.

## Materials and Methods

### *Populus euphratica* Natural Population and Seedling Propagation

**Figure 1:**
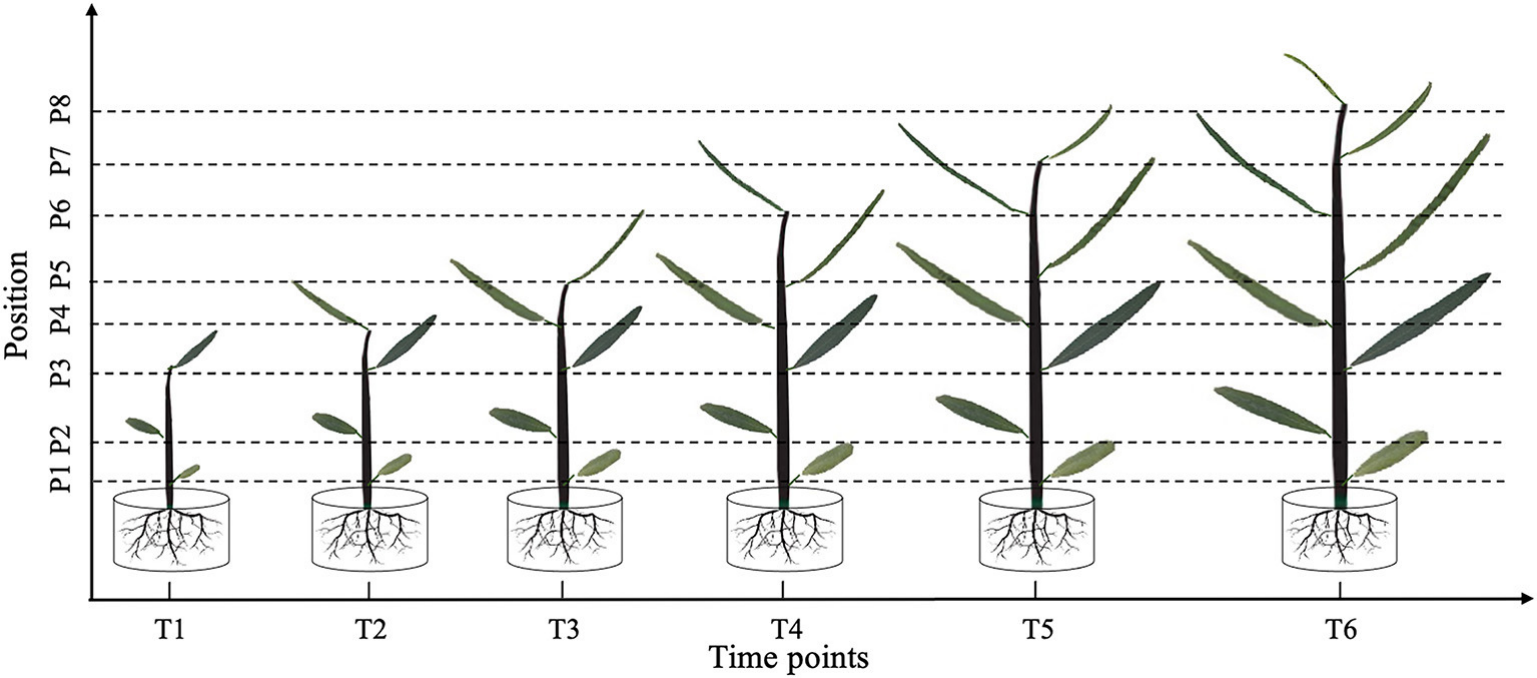
Leaf shape tracking strategy. T1–T6 indicate the time points with an equal time interval. At T1, leaves from the lower, middle, and upper part of the tree were selected for photographic tracking. At T2–T6, newly formed leaves from the upper part of the tree were selected for additional photographic tracking.

To construct the mapping population, seedlings of *P. euphratica* originally grown in the Tarim River Basin in Yuli County, Xinjiang Uygur Autonomous Region of China, were collected in October of 2017. Trees with good growth, and without pests or disease, were selected; 2-year-old dormant winter branches were then collected from the upper canopy of the trees. In total, 560 genotypes were collected to construct a natural population of *P. euphratica*, at a sampling interval of >50 m (Supplementray Figure 1).

The branches were hydroponically cultured in a greenhouse at Beijing Forestry University. Newly sprouted axillary buds from hydroponic branches were collected for further sterilization with 70% alcohol and 1% sodium hypochlorite solution. Using these starting materials, clonal seedlings were obtained through callus induction, shoot induction, and rooting culture. After leaves had been removed, stem segments including two axillary buds were cut into 2-cm segments, which were inoculated into 1/2 Murashige and Skoog (MS) callus induction medium supplemented with 0.2 mg/L N^6^-benzyladenine (6-BA), 0.5 mg/L α-naphthalene acetic acid (NAA), 30 g/L sucrose, and 7 g/L agar. The calli were subcultured three times to ensure their physiological status and obtain high and stable differentiation efficiency. When calli attained 2–3 cm in length, they were cut into 0.5- to 1-cm segments and transferred to MS supplemented with 0.4 mg/L 6-BA, 0.2 mg/L NAA, 0.05 mg/L brassinolide (BL), 20 g/L sucrose, and 7 g/L agar for bud induction. After 10 days, the newly induced buds (>3 cm in length) were separated and transferred to 1/2 MS medium supplemented with 8 g/L agar, 25 g/L sucrose, and 0.4 mg/L indole-3-butyric acid (IBA) for rooting. Due to the intrinsically weak regeneration capacity of *P. euphratica*, we harvested seedlings with 106 genotypes from among the 560 genotypes of the natural population. All of these culturing steps were conducted at 26°C under cool-white fluorescent light with a 16-h photoperiod and 1,500 lux. Complete plantlets (>5 cm) were cultured in 8 cm × 8 cm nutrient bowls containing vermiculite to harden the seedlings; the bowls were covered with transparent polythene bags to ensure maximum humidity. During the following year, all seedlings were transferred to large nutrient bowls containing a 1:1:1 ratio of vermiculite:perlite:soil and placed in a greenhouse at Beijing Forestry University for experiments and phenotype observation.

### Experimental Design

**Figure 2:**
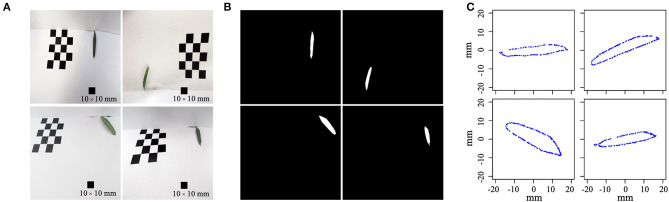
Diagram of the leaf shape reconstruction process. **(A)** Four original leaf images were randomly selected from a batch of images; each image was taken from a random angle. **(B)** Leaves were binarized on the raw image using the pixel coordinates of a fixed leaf. **(C)** Reconstructed leaf shapes, with two-dimensional coordinates along the leaf boundary, were calculated to reflect the size and morphology of the original leaf.

In March 2018, clonal seedlings with 106 genotypes showing normal development into complete plants were selected for the experiments. Among these, 20 genotypes had two replicate seedlings and the remaining 86 genotypes had 4–10 clonal replicates. To explore whether heterophylly is related to salt tolerance and whether salinity affects the variation of the heterophylly in *P. euphratica*, we used these materials to create a control group covering all 106 genotypes, and a salt treatment group covering 86 genotypes, which contained more clonal plants. According to a previous study (Jiang et al., 2020), the control and salt treatment groups were treated with clean water and 300 mM NaCl every 5 days. The NaCl concentration for the salt treatment group was determined according to the methods described in previous studies. Significant differences in the physiological activities of superoxide dismutase (SOD), catalase (CAT), peroxidase (POD), and malondialdehyde (MDA) between the control and salt treatment groups are shown in Supplementray Figure 2.

### Leaf Phenotypes and Image Processing

In June 18, 2018, after all the selected seedlings had experienced the hardening-off process for 3 months, we initially applied 300 mM NaCl to the salt treatment group. Leaf morphology was measured at different parts of the seedlings every 20 days, with a total of six leaf phenotype measurement periods throughout the growing season. Due to temporal and positional changes in *P. euphratica* heterophylly, images were used to fully track all morphological changes in the leaves. To avoid the pathogen resistance effect on heterophylly, leaves were daily managed to ensure their normal development. Leaves with all green colors from different growth positions at each period were selected for the investigation of phenotypes. The process of tracking leaf morphological changes over time is illustrated in Figure 1.

At time point T1, leaves at the bottom, middle, and top of each seedling were photographed *in vivo*, without removal. At subsequent time points, i.e., T2–T6, we tracked the morphology of these three leaves, as well as newly formed leaves at the top of the seedling. Because the leaf shape information was collected dynamically, we could not isolate the leaves for photographing. Therefore, we used a leaf reference image consisting of a fixed 1 × 1 cm grid printed on cardboard to standardize the leaf position and camera settings for each photograph (Figure 2). All photographs were taken using a Nikon Coolpix A10 camera with a minimum focal length. After completing the leaf imaging, we extracted a set of internal and external camera parameters using the CameraCalibrator module of MATLAB 2016a software (v9.0.0.341360; MathWorks, Natick, MA, United States). Based on the binarized shape contours converted from the RGB images, we calculated the contour coordinates of each leaf on the grid to determine its size and morphology with the aid of three-dimensional (3D) reconstruction. Based on the reconstructed 3D shapes, leaf phenotype traits such as length, width, and area were further extracted using an original MATLAB script.

### Principal Component Analysis (PCA) of Leaf Shape

To obtain the shape variation data from the reconstructed shape contours, we performed elliptic Fourier analysis to normalize the shape and, to remove any bias caused by size or rotation (Claude, 2008). Next, we performed PCA of the normalized shapes in the control and salt treatment groups using the *momocs* package in R v3.6.3 software (Bonhomme et al., 2014). The *PCcontrib* function was used to determine the contribution of each principal component (PC) to shape variation.

### ANOVA of Leaf Length

To examine the effects of genotype on leaf shape, we performed ANOVA. Traits were selected based on the top PCs identified through PCA. The phenotypic value (*y*) of each trait was expressed as follows:

(1) $$\begin{array}{l} y_{l|ijpt}\;=\;\mu \;+\;g_{i}\;+\;m_{j}\;+\;p_{p}\;+\;t_{t}\;+\;GPI_{ip|l}\;+\\ MPI_{jp|l}\;+\;GTI_{it|l}\;+\;MTI_{jt|l}\;+\;MGPI_{jip|l}\;+\;\varepsilon_{ijpt} \end{array}$$

where *l* indicates a given leaf of genotype *i* from treatment *j* at position *p* and time *t, g*_*i*_ is the genotype effect, *m*_*j*_ is the treatment effect, *p*_*p*_ is the position effect from the bottom to the top of the tree, *t*_*t*_ is the time effect, and ε_*ijpt*_ is the residual. The model contained an interaction term representing the genotype × position, treatment × position, genotype × time, treatment × time, and/or treatment × genotype × position interaction effects. ANOVA was performed using the *aov* function in R.

### SNP Genotyping and Population Structure

Using callus from each genotype in the natural population, we extracted genomic DNA using a plant genome DNA extraction system (Tiangen, Beijing, China). DNA purity was determined using a micro-ultraviolet light spectrophotometer (NanoDrop 2000C; Thermo Fisher Scientific, Waltham, MA, United States). The sequencing criterion was a 260-nm/280-nm optical density ratio (OD_260_/OD_280_) of 1.8–2.0. Whole-genome resequencing for all selected genotypes was performed (HiSeq platform; Illumina, San Diego, CA, United States) using a paired-end sequencing strategy (150 bp) and a 10 × sequencing depth. After the filtration of low-quality reads using the Burrows–Wheeler Aligner (BWA) software, clean reads were mapped to the reference genome (https://www.ncbi.nlm.nih.gov/search/all/?term=Populus%20euphratica; Ma et al., 2013) and then sorted using SAMtools software (Li et al., 2009). A total of 6,978,931 SNPs were detected using the Genome Analysis Toolkit (GATK) v3.0 software (Mckenna et al., 2010). The variants thus obtained were subjected to the bcftools filter to derive the final VCF file for genome-wide genotype recoding. Next, we applied the VCFtools software, with the following settings: -s LOWQUAL -i “%QUAL>20,” which retains a minimum allele frequency (MAF) > 0.01, missing genotype rate < 0.05, and Hardy–Weinberg equilibrium test *p* > 0.001 (Danecek et al., 2011). Finally, 241,990 high-quality SNPs were obtained for association analysis.

**Figure 3:**
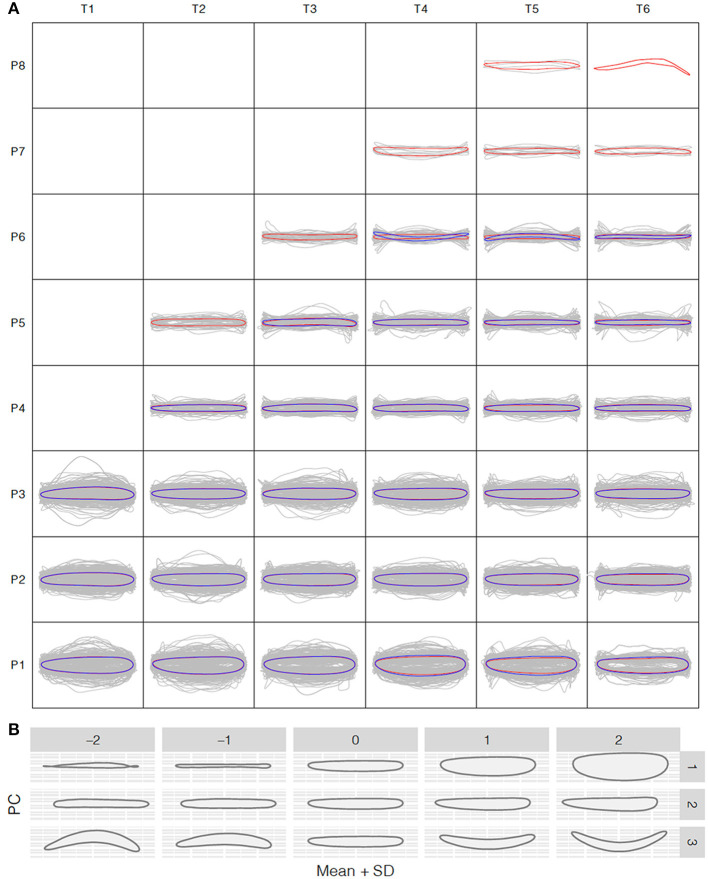
Shape variation analysis along the *P. euphratica* shoot axis. **(A)** Normalized shape profiles under control and salt stress conditions at T1–T6 (20-days intervals) and leaf positions P1–P8 (from bottom to top), controlled for size effects. Gray lines indicate the individual leaf shapes; red and blue contours indicate the average shapes calculated at each time and position for the control and salt stress groups, respectively. **(B)** Principal component analysis (PCA) of leaf shape. PC1–PC3 are the top PCs, cumulatively explaining 42.18, 72.56, and 93.41% of the total leaf shape variation in terms of the length/width ratio, lamina base shape, and shape curvature, respectively.

The structure of the natural population was estimated using fastSTRUCTURE software (Raj et al., 2014). We selected a value of *K* from 1 to 4 to determine the optimal number of subgroups and then calculated the maximum-likelihood value for each *K*. The optimal *K* value was determined using the Python script chooseK.py in fastSTRUCTURE. A maximum likelihood at *K* = 1 suggested no population structure with these 106 genotypes in the natural population of *P. euphratica* (Supplementray Figure 3).

### Statistical Model

We employed the allometric theory to model *P. euphratica* heterophylly at different positions along the shoot axis. For example, leaf length is relatively stable at the bottom of the tree throughout the growing season, whereas leaves in the middle and upper parts of the tree vary greatly in length with growth time. Therefore, allometric growth occurs at different positions within the tree and may be affected by plant hormones and leaf anatomical structure, which combine to form different local microenvironments. A position index can be used to represent these factors and their interactions at different parts within the tree.

### Position Index

Finlay and Wilkinson (1963) proposed a position index to quantitatively describe the microenvironment features influencing the leaf length at different positions within a plant. This index can be expressed as an average of trait values for all leaves at the target position. Given the important role of the developmental stage on the leaf length, the position index values change significantly during the growth process. We put forward the concept of position index in an allometric model because we consider the fact that leaves from different growth positions have different shapes and different growth speeds, which leads to unequal leaf length. Also because the pattern of leaf length across the distinct position was uncertain, the position index calculated from the mean leaf length at a certain position can roughly depict the position feature. Therefore, using the ascended-ordered position index, the heterophylly pattern of leaf length can be summarized within the allometric equation. Therefore, we propose a time-specific position index, expressed as follows:

(2) $$\begin{array}{l} P\left(t\right)=\left(P_{1}\left(t\right),P_{2}\left(t\right),\cdots ,P_{p}\left(t\right)\right)\\ \;=\left(y_{1}\left(t\right),y_{2}\left(t\right),\cdots ,y_{p}\left(t\right)\right) \end{array}$$

where *P*_*p*_ (*t*) = *y*_*p*_ (*t*) represents the average length at the *p*^th^ position from the bottom to the top at time *t*.

### Heterophylly Phenotype

Similarly, we described the longitudinal heterophylly phenotype of individual *i* measured at each position at time *t*, as follows:

(3) $$y_{i}\left(t\right)=\left(y_{i1}\left(t\right),y_{i2}\left(t\right),\cdots ,y_{ip}\left(t\right)\right)$$

where the number of elements within *y*_*i*_(*t*) vector varies with *t* = (1, 2, …, *T*) because seedlings have different numbers of leaves at different times. When *t* = 1, *y*_*i*_(*t*) contains three phenotype elements from the lower, middle, and upper parts of the tree at the initial time. When *t* = 5, *y*_*i*_(*t*) for the same individual also contains length data for newly developed leaves.

### Allometric Modeling of Heterophylly

Based on Equations (2) and (3), we assigned a longitudinal vector integrating length data at time *t* for a single individual as an independent phenotype vector. Vectors constructed for distinct time points were considered phenotype replicates, regardless of the number of leaves present at that time point. According to allometric theory, heterophylly can be modeled using a power equation, such that the changes in leaf length according to the position index at corresponding time *t* can be expressed as follows:

(4) $$\mu_{p}\left(t\right)=\alpha \;{\left(P_{p}\left(t\right)\right)}^{\beta }$$

This exponential power Equation (4) can provide a flexible fitness to the (quasi-)dynamic growth pattern, as many sigmoidal equations use time as *x-*axis, but this model can use position index as *x-*axis, which might be more accommodated to the real growth process. Second, though a simpler curve shape than sigmoidal equations, parameters α and β together can also combine to form more complex curve shapes, where α and β are the curve parameters for allometric growth. After logarithmic transformation, these two parameters describe the change rate and intercept for heterophilic development. Estimates of α and β can be used when discussing the important biological problems, such as the relationships between QTLs and positional variation in leaf length; thus, changes in α and β on the heterophylly curve indicate genotypic responses to environmental change.

### Genes Associated With Heterophylly

To detect genes regulating heterophylly, we performed the association analysis within the natural *P. euphratica* population using a genome-wide multivariate association mixed model (GMA, Ning et al., 2019). The GMA is a high-order non-parametric model that can be used to fit plant growth to any curve shape and to investigate traits throughout the dynamic growth process according to their intrinsic physiology without knowing the precise growth equation. The GMA considers covariate and kinship effects in association analyses using longitudinal data by incorporating a Q+K model and can be extended to unbalanced longitudinal data measured under heterophylly, for example, using the position index and corresponding leaf length data as *x* and *y* values, respectively.

We also applied functional mapping gene association methods (Wang et al., 2019). QQ-plot comparisons were used to identify reasonable association results for the power model. Assuming *J* genotypes at each SNP, the likelihood was expressed as follows:

(5) $$L\left(y\right)=\;\prod_{j=1}^{J}\left(\prod_{i=1}^{n_{j}}\left(f_{j}\left(y_{i}\left(1\right)\right)•f_{j}\left(y_{i}\left(2\right)\right)•\ldots •f_{j}\left(y_{i}\left(T\right)\right)\right)\right)$$

where *n*_*j*_ is the number of individuals carrying genotype *j* of an SNP, and *f*_*j*_(*y*_*i*_(*t*)) is the multivariate normal distribution density function of the length phenotype, integrating all positions of individual *i* at time *t*. The mean vector for *f*_*j*_(*y*_*i*_(*t*)) is expressed as follows:

(6) $$\begin{array}{l} \mu_{j}\left(t\right)=\left(\mu_{j1}\left(t\right),\;\mu_{j2}\left(t\right),\ldots ,\mu_{jp}\left(t\right)\right)=\\ \left(\alpha_{j}\;{\left(P_{1}\left(t\right)\right)}^{\beta_{j}},\alpha_{j}\;{\left(P_{2}\left(t\right)\right)}^{\beta_{j}},\ldots ,\alpha_{j}\;{\left(P_{p}\left(t\right)\right)}^{\beta_{j}}\right) \end{array}$$

where α_*j*_ and β_*j*_ are the heterophylly curve parameters specific to SNP genotype *j*. For the multivariate normal density function, the variance–covariance matrix Σ_*i*_(*t*) was equivalent to the leaf number of individual *i* at time *t*. The first-order structured antedependence [SAD(1)] model was used to model Σ_*i*_(*t*), as determined by two parameters, i.e., ϕ and *v*^2^ (Zhao et al., 2005), in R software (Code availability). After the parameters had been estimated and significant SNPs had been identified at the genome level, hypothesis testing was performed to identify SNPs that significantly regulate heterophylly variation, as follows:

(7) $$H_{0}:\left(\alpha_{j}\;\beta_{j}\right)=\left(\alpha \;\beta \right),for\;j=(1,2,\ldots ,J)$$

*H*_1_: At least one of the equalities in the H_0_ does not hold.

The log-likelihood ratio (LR) calculated between *H*_0_ and *H*_1_ was chi-square-distributed, and the converted *p-*value was compared with a permutation-determined threshold to determine the significance of the SNP. Next, we tested whether heterophylly curves for the same genotype differed significantly between the control and saline treatment groups. This test played a key role in screening the important saline-responsive heterophylly genes.

## Results

### Reconstruction of Leaf Shapes From Images

We recorded the leaf shapes throughout the growth season for each treatment using the method illustrated in Figure 2A. For each image, we used the CameraCalibrator module of MATLAB to calculate the camera position and distance to the grid as a 3 × 3 rotation matrix (*R*) and 3 × 1 translation vector (*T*). Using the camera focus length (*f*) and pixel coordinate system (*u*_0_*, v*_0_), we produced a 3 × 3 intrinsic matrix (*K*) at the pixel scale of the camera (*f*_*x*_, *f*_*y*_), derived as $\left(\begin{array}{ccc} f_{x} & \;0 & \;u_{0}\\ 0 & \;f_{y} & \;v_{0}\\ 0 & \;0 & \;1 \end{array}\right)$. Combining the intrinsic and external image parameters, we established an image projection matrix as *M* = *K*[*R T*] for each image, which was further used to multiply the exhausted 3D voxel to derive its two-dimensional (2D) projected coordinates on the image plane. Using the binarized leaf shape on the raw image (Figure 2B), all projected 2D coordinates were compared to the binarized shape contour, and coordinates that fell on the binarized leaf contour were selected to form the contour shape (Figure 2C).

The centroid of each resulting reconstructed shape overlapped the (0, 0) point of the image. Therefore, all shapes were aligned to contrast differences in size and morphology and to form an absolute reference for comparison. Overall, the detailed leaf shapes were well-recovered, with unbiased contours for quantitative extraction of size and morphology data. The reconstructed leaf shapes were further compared in terms of allometric leaf boundary expansion, with consideration of the effects of time and leaf position on leaf shape throughout the growth period. Following shape reconstruction, we obtained 3,677 and 1,780 shapes for the control and salt stress treatments, respectively, demonstrating successful non-invasive tracking of *in vivo* leaf shape in *P. euphratica* seedlings and the elimination of spatial effects on the images. Thus, our approach is feasible for future heteroblasty or heterophylly research.

### Shape Variation Analysis

To understand how leaf morphology changes with time and position, we normalized the 3,677 and 1,780 reconstructed leaf shapes for the control and salt stress treatment groups, respectively, to remove the size effect and rotation bias for shape comparison. We performed the normalized elliptic Fourier analysis to assign shapes to each time point and growth position for the control and salt stress treatment groups, and we obtained a mean contour of leaf morphology. Population-wide average shapes for the same growth position showed a similar appearance along the time axis (Figure 3A). However, at the same time point, larger fluctuations were observed in leaves from the bottom than from the top of the tree, with only long, strip-shaped leaves occurring near the crown. Thus, we observed an increase in the length-to-width ratio from the bottom to the top of the tree, suggesting greater variation in leaf function in a more complex environment. The length-to-width ratio was significantly different between apical lanceolate leaves (position ≥ P4) and the bottom three leaf positions. This pattern was observed in both treatment groups, despite the production of fewer leaves under salt stress, thus providing strong physiological support for heterophylly in *P. euphratica*. At positions P1–P3, there were no significant differences in normalized leaf shapes between the control and salt stress groups (Figure 3A), indicating that salt stress has less influence on leaf shape and a weaker heteroblasty effect.

To further explore the leaf shape variation across all positions and time points, we performed PCA using the normalized elliptic Fourier parameters. For all shapes (mean ± 2 SD), the cumulative contribution of the top three PCs explained 42.18%, 72.56%, and 93.41% of the total variance, with PC1–PC3 controlling the variance in the length-to-width ratio, leaf tip shape, and shape curvature, respectively (Figure 3B). PC1 clearly showed that the most morphological variation was caused by a variation in leaf length along the seedling axis.

### Leaf Length and Heterophylly

We performed ANOVA to evaluate the effects of all factors potentially acting on leaf length, e.g., salt stress, genotype background within the natural population, leaf position, growth, and all potential two- and three-factor interaction effects. All tested effects were found to be significant, except for the genotype × time point interaction (Table 1). Leaf position contributed the largest amount to the leaf length variation, followed by treatment and genotype, suggesting the necessity of inspecting genotypic variation at the allelic level.

**Table 1:** Results of ANOVA: genotype and position effects on *Populus euphratica* leaf length.

**Source**	***F***	***P*-value**
Treatment	111.905	<2e-16
Genotype	67.651	<2e-16
Position	271.071	<2e-16
Time point	26.956	<2e-16
Genotype × Position	6.532	<2e-16
Treatment × Position	9.241	9.26e-09
Genotype × Time point	0.843	0.99583
Treatment × Time point	4.257	0.00073
Treatment × Genotype × Position	10.130	<2e-16

In the control group, the leaf length was stable at positions P1 and P2 throughout the growth period, but differed significantly between the first two time points at positions P3–P8, indicating that new leaves at these positions attained their shape rapidly and then continued to increase in length throughout the growth period. Along the vertical axis, leaves at the subterminal position were longest. These spatiotemporal heterophylly patterns were maintained under salt stress; compared with the control group, positions P1–P3 had 8.29 and 6.53% shorter and 1.25% longer leaves, respectively. At positions P4–P6, a substantial difference in length was observed between the first two time points, when new leaves first appeared, but this difference decreased over time, suggesting a balance between leaf growth and salt tolerance.

### Mapping Heterophylly QTLs as a Function of Position Index

**Figure 4:**
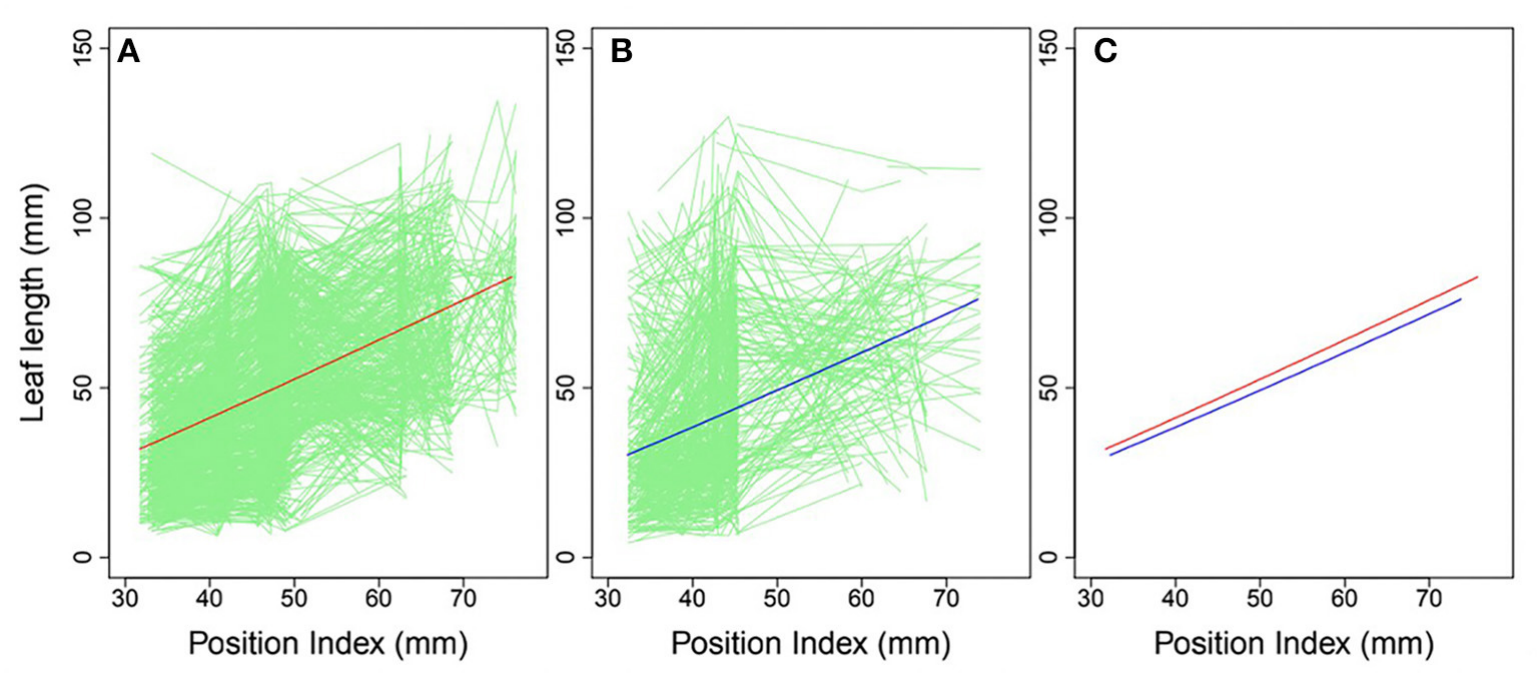
Heterophylly curve of leaf length vs. position index. Green lines in **(A,B)** are individual heterophylly curves for the control and salt stress groups, and red and blue lines indicate their mean heterophylly curves. **(C)** Comparison of the two mean curves.

Heterophylly curves were plotted as the longitudinal leaf length at each position against the position index, with replicates for each time point (Figure 4). Allometric growth is described by the power equation given in Equation (4) and provided a good fit to leaf length heterophylly in both the control and salt stress groups. Although fewer leaves were initiated at positions P4–P8 during the later growth stages under salt stress, leaf responses varied widely among position index values. At the population level, the average curve for the control group had values of α = 0.7314 and β = 1.0926 (Figure 4A), while the average salt stress curve had values of α = 0.6246 and β = 1.1165 (Figure 4B). Despite its slightly higher slope, the salt stress heterophylly equation had generally lower values, demonstrating that heterophylly was sensitive to salt stress. The salt stress group had a narrower range of position index values than the control group (Figure 4C), suggesting a narrower range of leaf length along the seedling axis.

**Figure 5:**
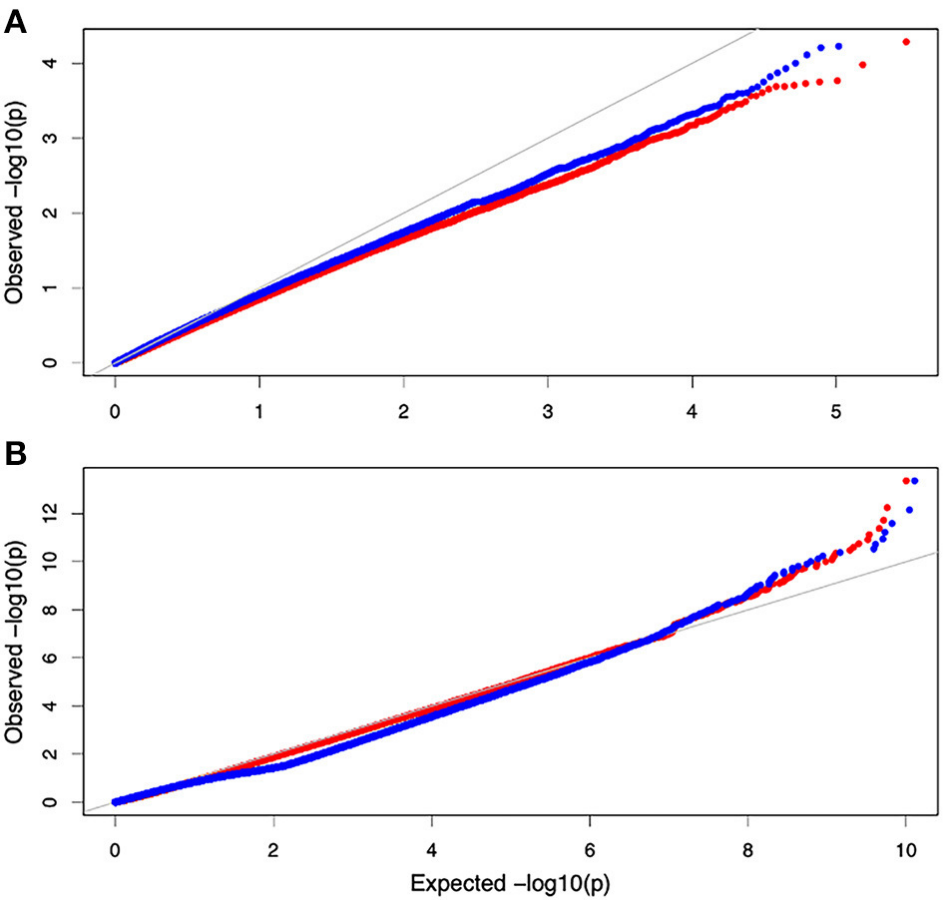
QQ plot of the gene association analysis results. **(A)** QQ plot derived from the genome-wide multivariate association mixed model (GMA) results. **(B)** Distribution of *p*values derived from the functional mapping model.

**Figure 6:**
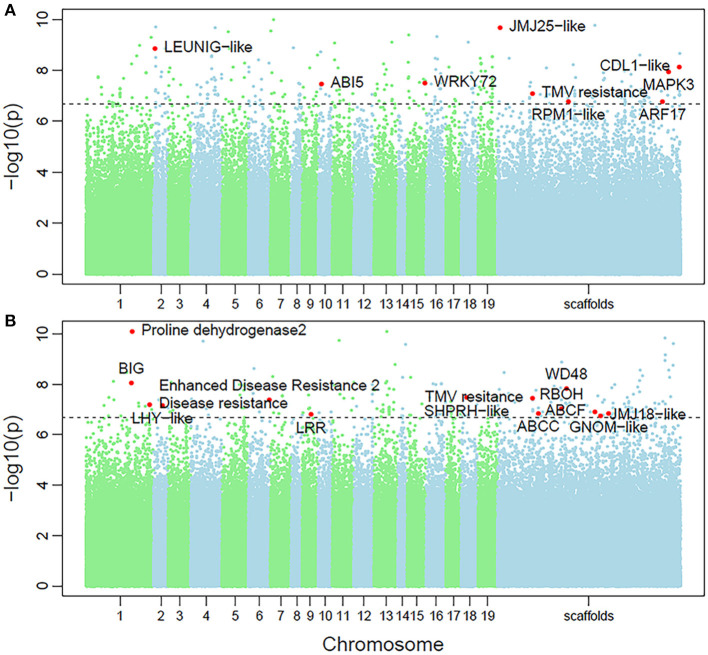
Manhattan plots of genome-wide association of heterophylly trait results for the **(A)** control and **(B)** salt stress groups. Dotted line indicates the 5% significance threshold.

Non-parametric GMA modeling of the longitudinal heterophylly curve resulted in a set of low, segregated *p-*values compared with the expected distribution, for both the control and salt stress groups (Figure 5A), indicating insufficient power to detect the causal SNPs of heterophylly. Because permutation tests were used to determine the functional mapping threshold, most *p-*values approached the uniform distribution, as expected (Figure 5B), except that the QQ-plot had an upturned tail. Based on the multiplicative likelihood function and functional mapping for both control and salt stress conditions, we estimated coefficients α and β for each SNP genotype and used them to evaluate the effects of significant SNPs on the shape of the heterophylly curve. We used the mapping model to scan the genome-wide SNPs throughout all chromosomes and other scaffolds for QTLs; the LR identified a total of 349 and 214 SNPs that were highly associated with heterophylly curves for control and salt stress conditions, respectively (Figures 6A,B). Significant SNPs were distributed sporadically throughout all chromosomes and unanchored scaffolds and were annotated to 171 and 134 genes; 66 and 57 protein products were found to contain them (Supplementray Tables 1, 2). However, among these significant genes, none were common to both treatment groups. Through a detailed functional annotation, including Gene Ontology (GO) enrichment analysis, we examined genes with key functions and classified them into several categories, including leaf morphogenesis, auxin response, immune system, ABA response, stress resistance, and anatomical structure development (Table 2).

**Table 2:** Functional categories of significant genes associated with heterophylly in *P. euphratica*.

**Treatment**	**Gene classes**	**Genes annotation**	**GO term**	**GO ID**	***P*-value**
CK	Stress resistance	WRKY transcription factor 72 (LOC105141593, LOC105116025), (LOC105129774, LOC105130544, LOC105117132, LOC105117711, LOC105121333)	Defense response	GO:0006952	0.0069
		ABSCISIC ACID-INSENSITIVE 5-like protein 2 (LOC105137304), (LOC105112451, LOC105129809, LOC105116181, LOC105112539)	Response to abscisic acid	GO:0009737	0.0049
		Serine/threonine-protein kinase CDL1-like (LOC105116025, LOC105117132), (LOC105117711, LOC105121333)	Immune system process	GO:0002376	0.0012
		Lysine-specific demethylase JMJ25-like (LOC105108522), (LOC105111440, LOC105137665)	Histone modification	GO:0016570	0.0074
		Mitogen-activated protein kinase 2-like (LOC105112451), (LOC105112339, LOC105129809, LOC105128439, LOC105137304, LOC105116181)	Response to hormone	GO:0009725	0.0092
	Auxin-related genes	Auxin response factor 17-like (LOC105112339), (LOC105112451, LOC105116181, LOC105137304)	Hormone-mediated signaling pathway	GO:0009755	0.0213
	Leaf shape related	Transcriptional corepressor LEUNIG-like (LOC105125785), (LOC105135597, LOC105116181, LOC105112339, LOC105111440)	Shoot system development	GO:0048367	0.0899
	Disease resistance	Disease resistance protein RPM1-like (LOC105117711), (LOC105116025, LOC105117132, LOC105121333)	Immune response	GO:0006955	0.0062
		TMV resistance protein N-like (LOC105109520), (LOC105117132, LOC105137304, LOC105121333, LOC105141593, LOC105116181, LOC105128601, LOC105111754, LOC105129809, LOC105128439, LOC105112339, LOC105132765, LOC105116025, LOC105117711, LOC105108966, LOC105112539, LOC105112451, LOC105129774, LOC105130544, LOC105112900, LOC105109520, LOC105108094, LOC105110122)	Response to stimulus	GO:0050896	0.0009
Salt	Stress resistance	Probable leucine-rich repeat receptor-like protein kinase At1g35710 (LOC105126665), (LOC105130009, LOC105125089)	Defense response	GO:0006952	0.4145
		Probable LRR receptor-like serine/threonine-protein kinase At1g07650 (LOC105107495), (LOC105126240, LOC105123321)	Response to osmotic stress	GO:0006970	0.2232
		Probable LRR receptor-like serine/threonine-protein kinase At1g53440 (LOC105136522), (LOC105126665, LOC105115456, LOC105114517, LOC105138604, LOC105129804, LOC105139638, LOC105121124)	Kinase activity	GO:0016301	0.0009
		E3 ubiquitin-protein ligase SHPRH-like (LOC105107433), (LOC105142410, LOC105110458, LOC105134419)	Ubiquitin ligase complex	GO:0000151	0.0081
		WD repeat-containing protein 48 (LOC105110458), (LOC105126665, LOC105126240, LOC105123276, LOC105124653, LOC105115456, LOC105131169, LOC105127854, LOC105110458, LOC105111425, LOC105109520, LOC105130122, LOC105117107, LOC105126350, LOC105112394, LOC105111615, LOC105142410, LOC105130009, LOC105129804, LOC105111272)	Regulation of biological process	GO:0050789	0.0003
		Respiratory burst oxidase homolog protein (LOC105110346), (LOC105126665, LOC105110346, LOC105117107, LOC105126240, LOC105133021, LOC105109520, LOC105124653, LOC105115456, LOC105123276, LOC105127854, LOC105111425, LOC105111037, LOC105140060, LOC105125089, LOC105123321, LOC105142410, LOC105130009, LOC105111272, LOC105126350)	Response to stimulus	GO:0050896	0.0015
		ABC transporter C family member 2-like (LOC105116788), (LOC105132765, LOC105126665, LOC105116788, LOC105111194, LOC105111425)	Transmembrane transport	GO:0036080	0.0120
		ABC transporter F family member 1-like (LOC105111194), (LOC105116788, LOC105132765, LOC105126665, LOC105111425, LOC105132765)	Transmembrane transport	GO:0036080	0.0120
		Lysine-specific demethylase JMJ18-like (LOC105111272), (LOC105140060, LOC105123276, LOC105111425, LOC105117107, LOC105126350, LOC105123321, LOC105126240)	Response to abiotic stimulus	GO:0009628	0.0192
		Proline dehydrogenase 2 (LOC105123321), (LOC105126240, LOC105123321)	Response to osmotic stress	GO:0006970	0.2232
	Auxin-related genes	Auxin transport protein BIG (LOC105123276), (LOC105142410, LOC105126240, LOC105140060)	Response to auxin	GO:0009733	0.0037
		Protein LHY-like (LOC105126240), (LOC105140060, LOC105123276, LOC105111425, LOC105117107, LOC105126350, LOC105123321, LOC105111272)	Response to abiotic stimulus	GO:0009628	0.0192
		ARF guanine-nucleotide exchange factor GNOM-like (LOC105111425), (LOC105123276)	Auxin polar transport	GO:0009926	0.0087
	Leaf shape related	Leucine-rich repeat receptor-like protein kinase (LOC105126665), (LOC105130009, LOC105125089)	Defense response	GO:0006952	0.4145
	Disease resistance	Protein ENHANCED DISEASE RESISTANCE 2-like (LOC105134415), (LOC105126665, LOC105130009, LOC105125089, LOC105126665)	Immune system process	GO:0002376	0.4908
		TMV resistance protein N-like (LOC105109520, LOC105111238), (LOC105126665, LOC105110346, LOC105117107, LOC105126240, LOC105124653, LOC105115456, LOC105123276, LOC105127854, LOC105111425, LOC105111037, LOC105140060, LOC105125089, LOC105123321, LOC105142410, LOC105130009, LOC105111272, LOC105126350)	Response to stimulus	GO:0050896	0.0015
		Probable disease resistance protein At4g27220 (LOC105125089), (LOC105126665, LOC105130009)	Defense response	GO:0006952	0.4145

**Figure 7:**
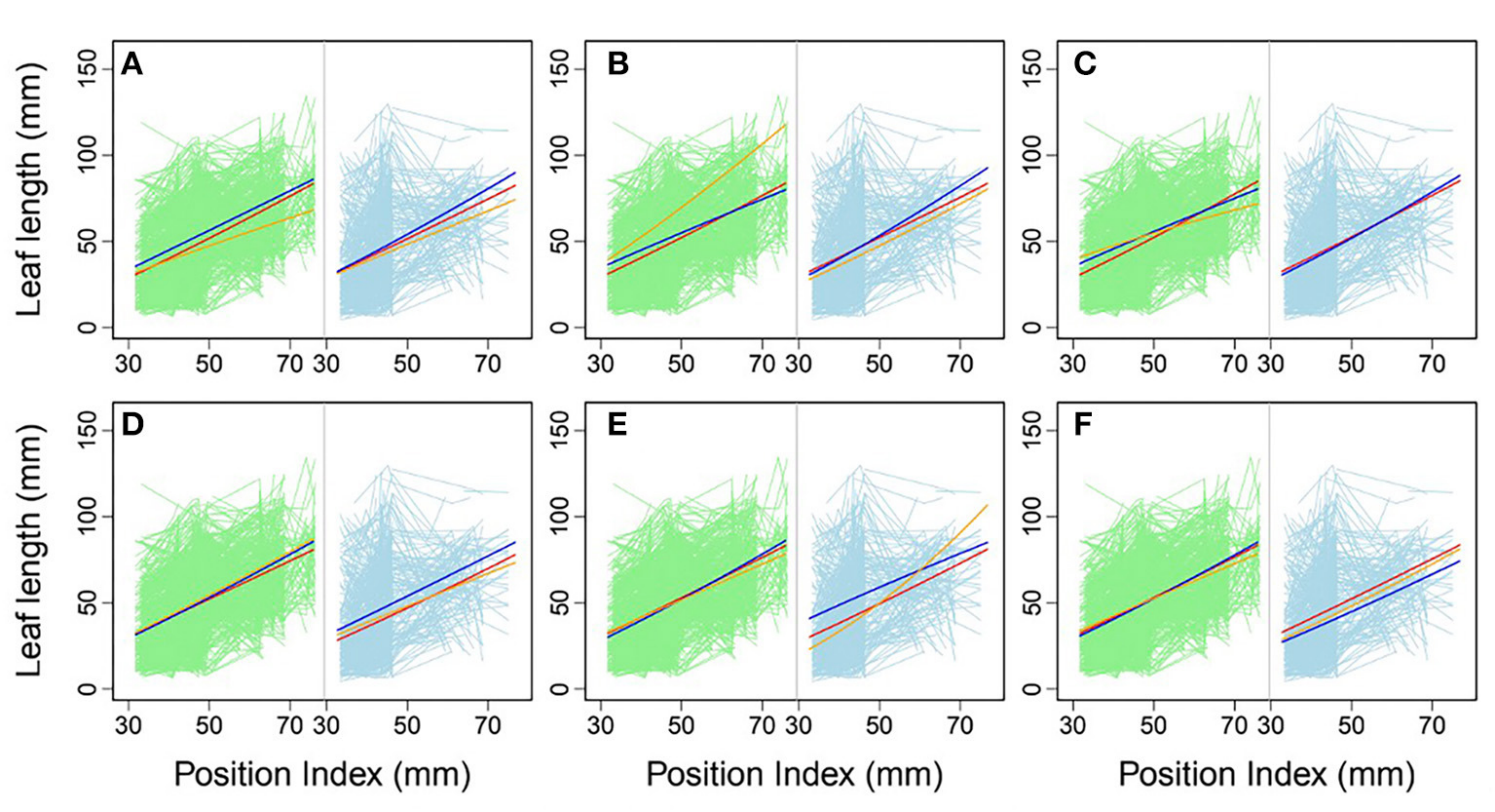
Comparison of allelic heterophylly curves with significant single-nucleotide polymorphisms (SNPs) between the **(A–C)** control and **(D–F)** salt stress groups. Green and light blue lines indicate individual heterophylly curves vs. position index for the control and salt stress groups, respectively. Thick dark blue, red, and orange lines indicate three different heterophylly genotypes.

Using allometric equations for each significant SNP, we plotted the genotype-dependent heterophylly curves indicating the relationship between leaf length and position index over time as development trajectories. For example, Figure 7 shows the SNP genotype-dependent difference between the heterophylly curves of the control and salt stress groups. At six selected significant SNPs, different genes affected the heterophylly pattern. SNPs on the *ABSCISIC ACID-INSENSITIVE 5-LIKE* (*ABI5*) (Figure 7A), *WRKY72* (Figure 7B), and *AUXIN RESPONSE PROTEIN 17-LIKE* (*ARF17*) genes (Figure 7C) exerted significant effects on the reaction norm of leaf length under control conditions, with differences between the effects of *ABI5* and *WRKY72* increasing along the vertical shoot axis. *ARF17* exhibited a crossover effect among allelic genotypes. Most of these genes showed an environment-specific expression, with genotypes diverging under one condition and being silenced under the other, suggesting an SNP–environment interaction. For example, genotypes of the *F-box, RESPIRATORY BURST OXIDASE HOMOLOG PROTEIN H* (*RBOH*), and *DISEASE RESISTANCE PROTEIN* genes (Figures 7D–F) showed varying heterophylly curve shapes under salt stress, but none showed a diverging pattern under control conditions. The differences in effect among alleles depended on the position of the heterophylly curve inflection point. Under salt stress, the effect of *ABI5* increased with position index, whereas that of the *F-box* gene remained stable (Figures 7A,D). Two genotypes of the *RBOH* gene expressed a decreasing allelic curve difference, whereas a third genotype expressed the inverse pattern, with smaller leaf length at lower position indices and larger length differences at higher positions along the shoot (Figure 7E).

### Heritability

**Figure 8:**
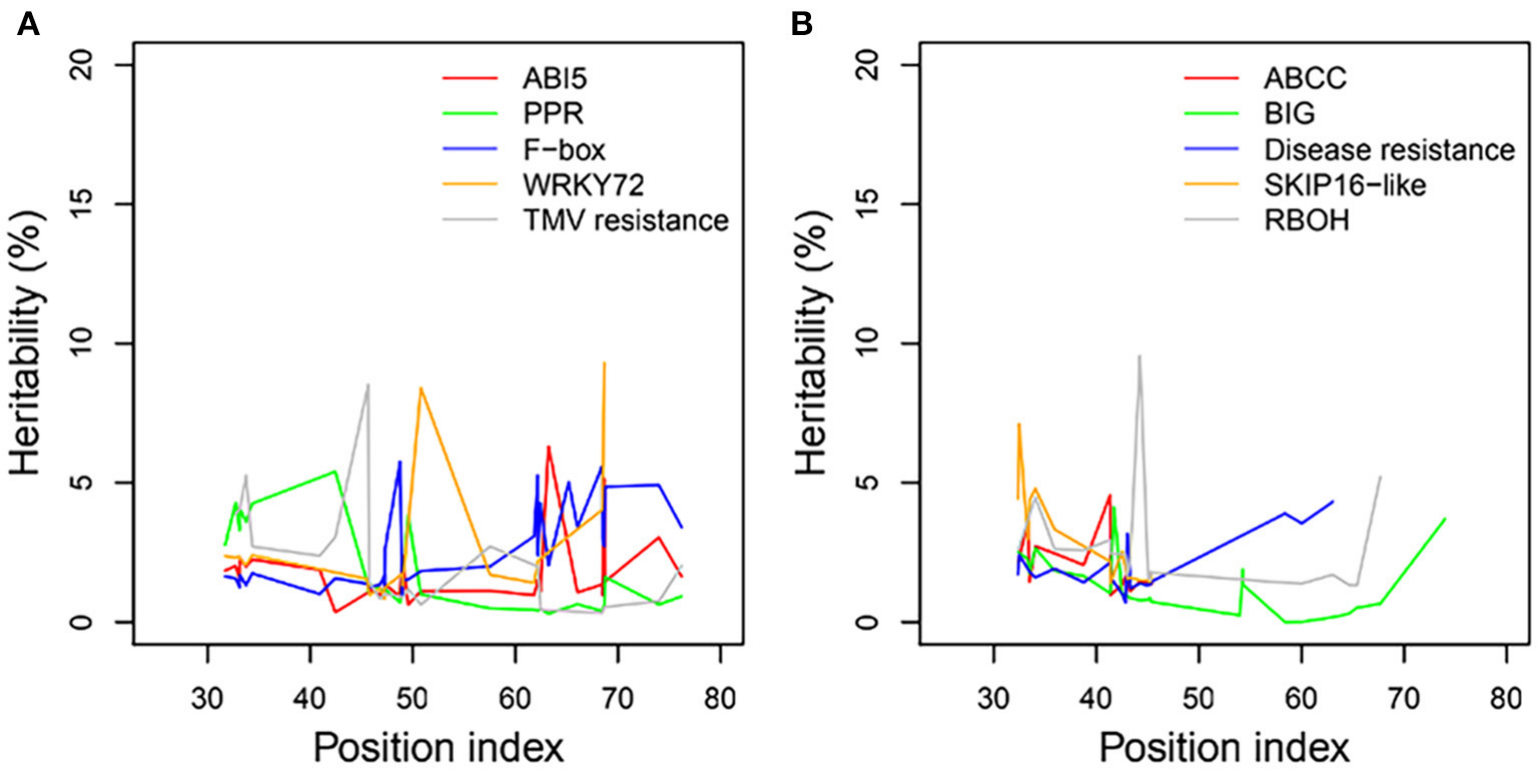
Heritabilities of significant genes regulating heterophylly for control and salt stress condition. **(A,B)** List the heritabilities of five different genes with different position index, respectively.

We examined the genetic variance components of significant genes classified into key functional categories at each position along the shoot axis by calculating their point-wise heritability. The overall heritability among all position indices was ~5%; there was some variation among position indices, and heritability did not always increase with the position index (Figures 8A,B). Heritability values may have been slightly overestimated due to the small sample sizes in this natural population. The mean heritability values for all positions were 1.75, 1.74, 2.53, 2.54, and 1.97% for genes coding *ABI5, PPR, F-box, WRKY72*, and *TMV RESISTANCE* protein, respectively, under control conditions, and 1.96, 1.31, 1.97, 2.74, and 2.63% for ATP-binding cassette subfamily C (*ABCC*), auxin transport protein *BIG, DISEASE RESISTANCE*, F-box protein *SKIP16-like*, and *RBOH*, respectively, under salt stress.

## Discussion

Leaf shape plays an important role in maintaining plant function and has a profound influence on plant environmental adaptability. Heterophylly and heteroblasty are central concepts in botany that describe the variation in leaf function (Winn, 1999; Zotz et al., 2011). Heterophylly promotes plant adaptation to endogenous and exogenous microenvironments, in processes that can involve leaf shape or size transitions along a single shoot. Quantitative genetic analyses are powerful tools to elucidate the genetic processes underlying heterophylly. In this study, we mapped heterophylly functions to saline tolerance in a natural population of *P. euphratica*.

We found that heterophylly patterns differed widely between juvenile and mature leaves, with adult *P. euphratica* leaves exhibiting many distinct shapes. *P. euphratica* is a model tree species for stress resistance because it survives frequently harsh environments from germination to maturity within its natural distribution. We hypothesized that heterophylly patterns would differ between control and saline stress conditions within the same *P. euphratica* population, and we expected that differences in continuous heterophylly patterns between groups of propagated clones in both conditions would allow us to determine whether salinity affects heterophylly (heterophilic plasticity) or whether heterophylly is an intrinsic property of saline tolerance during *P. euphratica* development.

We first conducted a tissue culture of *P. euphratica*, because regeneration capacity differs significantly among lines (Ferreira et al., 2009; Zhang et al., 2020). Because *P. euphratica* is a tree species with difficult tissue regeneration capacity, its branch cuttings cannot take root under the normal and extreme environment. To induce more clonal seedlings for each line in the natural population, steps of callus induction culture are necessary and are more feasible to generate seedling plantlet, though the callus induction using explants and the shoot or root induction process using callus had been complicated. Population-level callus, shoot, and root induction were conducted to propagate 106 lines of clonal seedlings, to provide a solid foundation for examining the relationship between salt tolerance and leaf phenotype. We tracked leaf shapes at different growth positions over time using a newly developed, non-invasive, and cost-effective tracking method involving leaf photography *in vivo* against a reference grid, to calculate 3D coordinates along the leaf boundary. The Procrustes-aligned shapes were normalized for spatiotemporal comparability, to facilitate heterophylly and heteroblasty research.

Next, we conducted the functional mapping of the quasi-dynamic trait of leaf length, which was associated with position index at the population level, to construct heterophylly curves. This approach simplifies mathematical expressions for complex traits. The annotation results of the control group (Table 2) supported the findings of a previous study indicating that *AUXIN RESPONSE FACTOR17-LIKE* (*ARF17*) plays a role in normal leaf elongation (Okushima et al., 2005). The detection of *ABSCISIC ACIS-INSENSITIVE 5-like* (*ABI5*) was consistent with the role of ABA in regulating heterophylly (Wanke, 2011). Other genes were found to significantly regulate defense, stress, and innate immune responses, including *WRKY72*, TMV resistance protein, disease resistance protein *RPM1-LIKE*, and the *MAPK3* gene. Both *ABI5* and *MAPK3* were found to be involved in the reactive oxygen species (ROS) pathway mediating responses to osmotic stresses, including salt stress (Deinlein et al., 2014; Julkowska and Testerink, 2015). *WRKY* functions in linking stress sensing to many tolerance responses (Phukan et al., 2016). Immune resistance gene expression may be induced by subordinate coordination of stress responses, since several studies have demonstrated their roles in disease resistance in many salt tolerance genes *via* pleiotropy (Chen and Guo, 2008, Chen et al., 2015). These findings suggest a role of innate higher expression of stress-responsive genes in heterophylly regulation, which further suggests a potential genomic background for heterophylly that differs greatly from those of other tree species.

Likely due to the smaller salt stress group sample size, caused by the difficulty of regenerating *P. euphratica*, the GWAS results showed no overlap of significant genes between the two treatment conditions. Genes exhibiting similar functions between both groups included the auxin export protein *BIG*, as well as TMV and disease resistance proteins. Many stress-related genes were detected, including *ABCC2* and ABC transporter F family member (*ABCF*), which function in stomata regulation and/or detoxification of stomatal guard cells, as well as stress response regulation (Kimball, 1999, Klein et al., 2006, Verrier et al., 2008). *RBOH*, which engineers ROS signaling, further contributes to stress tolerance (Suzuki et al., 2011). The large number of stress-regulating genes found in this study were consistent with the roles in the saline response. These findings of balanced regulation of auxin-related genes for normal leaf growth, large numbers of stress-related genes in the salt stress group, and altered heterophylly curves improve our understanding of continuous heterophylly patterns and their roles in salt tolerance in *P. euphratica*.

## Data Availability Statement

The code and shell script that support the findings of this study are available from https://github.com/YaruFu01/leafQTL or can be requested from the corresponding author.

## Author Contributions

YF and LJ derived the model. FL and SM participated in the field management of experimental materials, they also led the phenotype investigation, and data collection. MY performed data analyses and wrote the manuscript. RW conceived of the idea of the overall investigation. All authors contributed to the article and approved the submitted version.

## Conflict of Interest

The authors declare that the research was conducted in the absence of any commercial or financial relationships that could be construed as a potential conflict of interest.
