# Supplementary material for: Heterophylly Quantitative Trait Loci Respond to Salt Stress in the Desert Tree Populus euphratica
Source: Front Plant Sci. 2021 Jul 15;12:692494. doi: 10.3389/fpls.2021.692494 (PMC8321784; doi:10.3389/fpls.2021.692494)

**Figure S1**. Map of the sampling site comprising a natural population of *Populus euphratica* in Xinjiang, China. Sampling location were isolated using four different colors, as the sampling density is relatively high on the large-scale map.


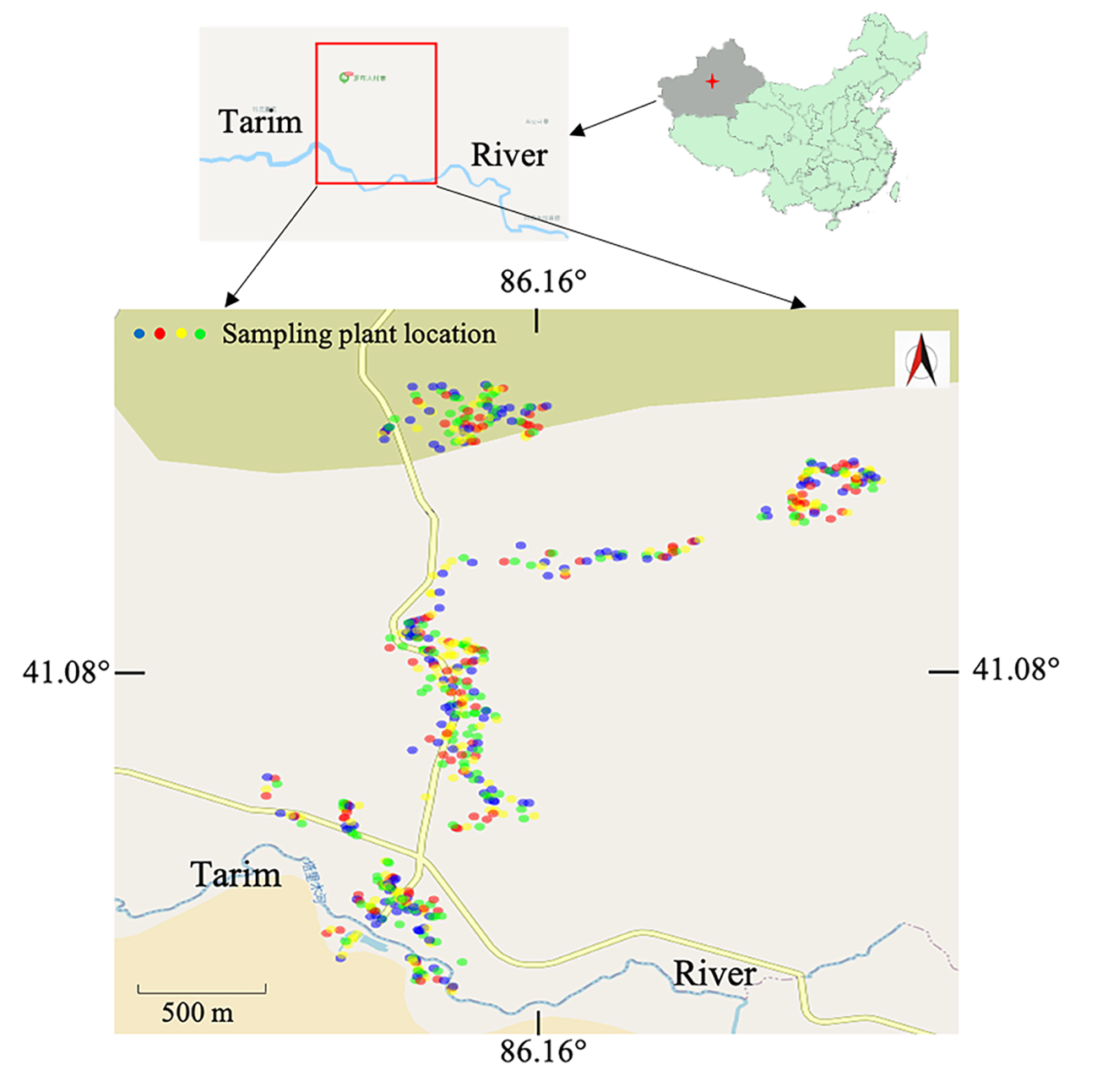

Supplement: Supplementary file 1 [file Data_Sheet_1.zip › Figure S1.DOCX]
