# Supplementary material for: Heterophylly Quantitative Trait Loci Respond to Salt Stress in the Desert Tree Populus euphratica
Source: Front Plant Sci. 2021 Jul 15;12:692494. doi: 10.3389/fpls.2021.692494 (PMC8321784; doi:10.3389/fpls.2021.692494)

**Figure S2**. The dynamic enzymatic activities with superoxide dismutase (SOD), catalase (CAT), peroxidase (POD) and malondialdehyde (MDA) at different time points under salt-free (CK) and salt condition (Salt). * denote the significant difference level at 0.05.


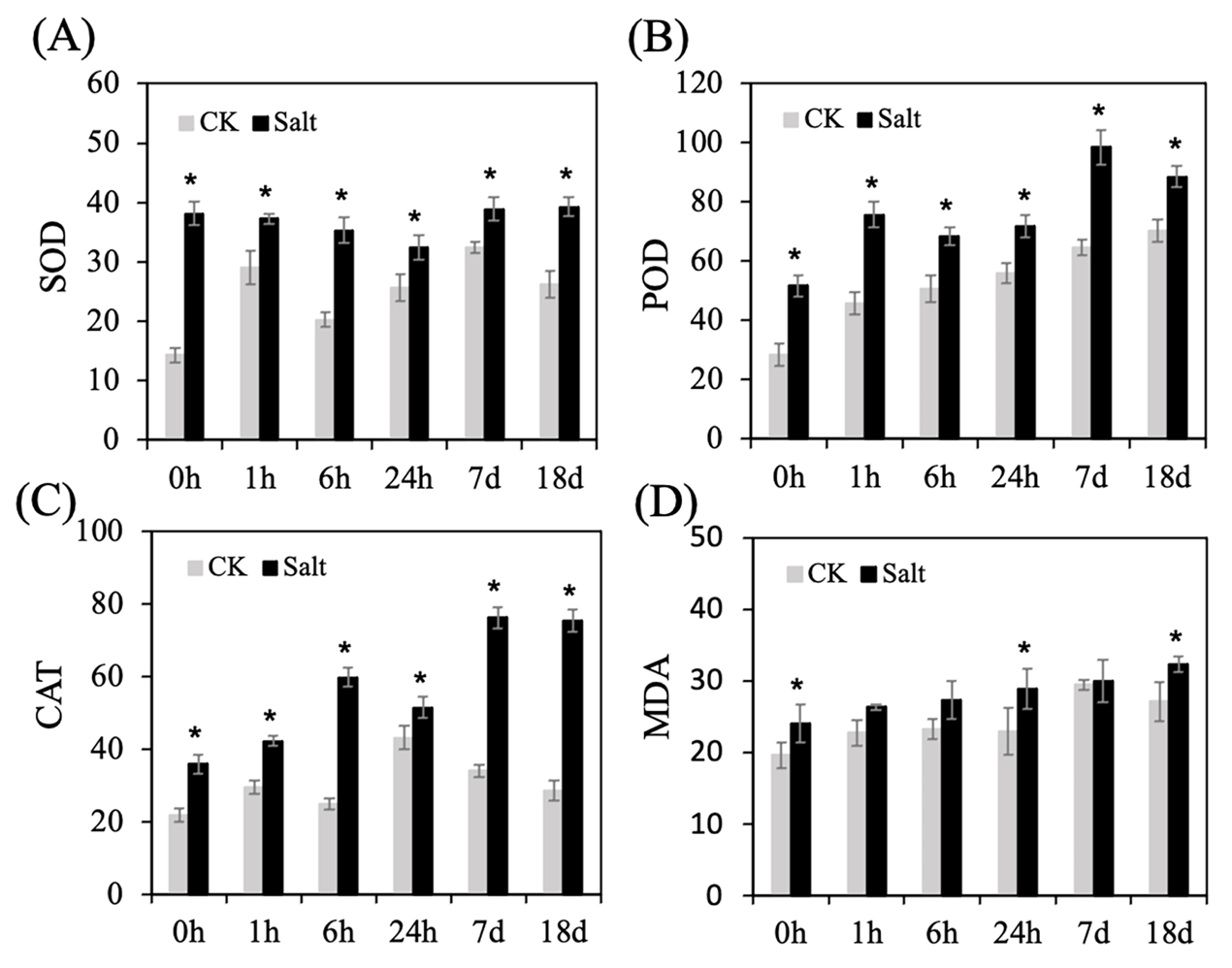

Supplement: Supplementary file 1 [file Data_Sheet_1.zip › Figure S2.DOCX]
