# Supplementary material for: Heterophylly Quantitative Trait Loci Respond to Salt Stress in the Desert Tree Populus euphratica
Source: Front Plant Sci. 2021 Jul 15;12:692494. doi: 10.3389/fpls.2021.692494 (PMC8321784; doi:10.3389/fpls.2021.692494)

**Figure S3**. Likelihood value against each *K*. For different *K*, the likelihood were calculated, red triangle indicates a best structure at *K* = 1. *K* means the number of subgroups.


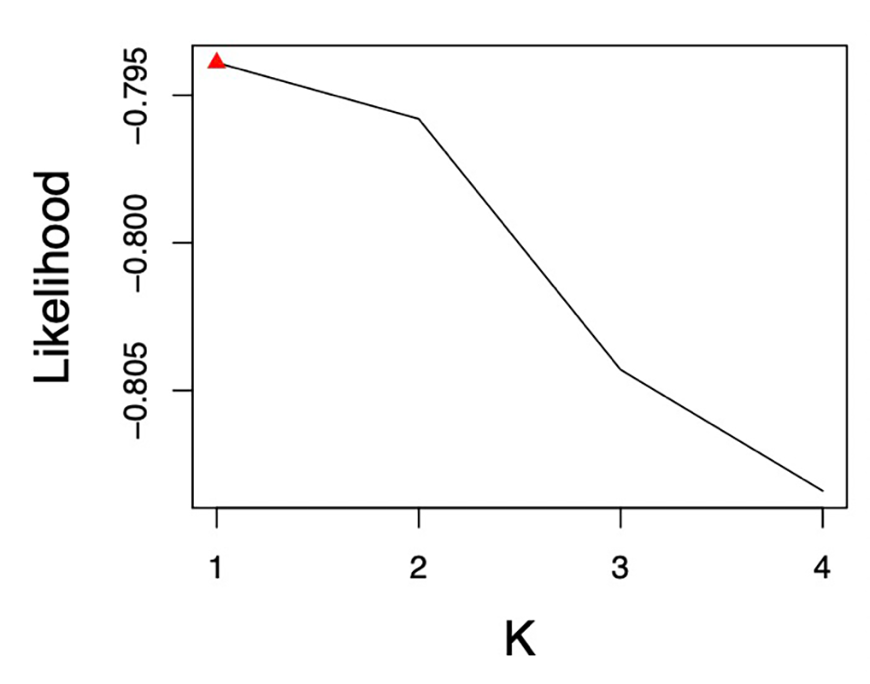

Supplement: Supplementary file 1 [file Data_Sheet_1.zip › Figure S3.DOCX]
