# Supplementary material for: Heterophylly Quantitative Trait Loci Respond to Salt Stress in the Desert Tree Populus euphratica
Source: Front Plant Sci. 2021 Jul 15;12:692494. doi: 10.3389/fpls.2021.692494 (PMC8321784; doi:10.3389/fpls.2021.692494)
Supplement: Supplementary file 1 [file Data_Sheet_1.zip › Table S1.DOCX]

**Table S1**. Gene information of significant SNPs for CK condition.

| Number | Chromosome ID | Distribution | Gene ID | Annotation |
| --- | --- | --- | --- | --- |
| 3504 | NW_011499846.1 | gene | LOC105131560 | UPF0481 protein At3g47200-like |
| 10977 | NW_011499850.1 | exon | LOC105121333 | protein P21-like |
| 13060 | NW_011499852.1 | gene | LOC105121714 | probable E3 ubiquitin-protein ligase |
| 15792 | NW_011499854.1 | exon | LOC105122543 | pentatricopeptide repeat-containing protein At4g33170-like |
| 16806 | NW_011499855.1 | exon | LOC105122795 | F-box protein At3g54460 |
| 20327 | NW_011499857.1 | gene | LOC105123564 | pre-rRNA-processing protein TSR2 |
| 24533 | NW_011499860.1 | gene | LOC105124664 | KH domain-containing protein At4g18375-like |
| 26129 | NW_011499862.1 | exon | LOC105125203 | ATP-citrate synthase alpha chain protein 2-like |
| 26551 | NW_011500662.1 | gene | LOC105115899 | protein disulfide-isomerase 5-4-like |
| 27953 | NW_011499864.1 | exon | LOC105125675 | CLIP-associated protein%2C transcript variant X1 |
| 28090 | NW_011499864.1 | gene | LOC105125785 | transcriptional corepressor LEUNIG-like |
| 29568 | NW_011500265.1 | exon | LOC105112900 | vicianin hydrolase-like |
| 40136 | NW_011499876.1 | exon | LOC105128440 | survival of motor neuron-related-splicing factor 30 |
| 40156 | NW_011499876.1 | gene | LOC105128439 | cullin-associated NEDD8-dissociated protein 1 |
| 41206 | NW_011499877.1 | gene | LOC105128601 | mannose-1-phosphate guanyltransferase alpha |
| 41221 | NW_011499877.1 | exon | LOC105128603 | pentatricopeptide repeat-containing protein At1g74900 |
| 46553 | NW_011499883.1 | gene | LOC105129774 | serine/threonine-protein kinase TOR-like |
| 46885 | NW_011499883.1 | gene | LOC105129809 | zinc finger protein-like 1 homolog |
| 48643 | NW_011499885.1 | gene | LOC105130152 | DNA polymerase I A%2C chloroplastic/mitochondrial-like |
| 48693 | NW_011499885.1 | gene | LOC105130180 | ankyrin repeat-containing protein At5g02620-like |
| 48842 | NW_011499885.1 | gene | LOC105130223 | G-type lectin S-receptor-like serine/threonine-protein kinase At4g27290 |
| 50920 | NW_011499887.1 | gene | LOC105130544 | probable protein phosphatase 2C |
| 51615 | NW_011499888.1 | exon | LOC105130645 | regulator of nonsense transcripts UPF3-like |
| 51628 | NW_011499888.1 | gene | LOC105130646 | putative methylesterase |
| 52511 | NW_011499889.1 | exon | LOC105130952 | pentatricopeptide repeat-containing protein At4g17616-like |
| 54120 | NW_011499891.1 | exon | LOC105131296 | acyltransferase-like protein At1g54570 |
| 57750 | NW_011500694.1 | gene | LOC105116025 | serine/threonine-protein kinase CDL1-like |
| 62126 | NW_011499899.1 | gene | LOC105132549 | inactive protein RESTRICTED TEV MOVEMENT 2-like |
| 63610 | NW_011499901.1 | gene | LOC105132764 | peroxisomal nicotinamide adenine dinucleotide carrier-like |
| 63681 | NW_011499901.1 | gene | LOC105132765 | UDP-galactose transporter 2 |
| 64899 | NW_011499902.1 | gene | LOC105132982 | HEAT repeat-containing protein 5B |
| 71226 | NW_011501509.1 | exon | LOC105117984 | GPCR-type G protein 2-like |
| 71345 | NW_011499910.1 | exon | LOC105133812 | pentatricopeptide repeat-containing protein At1g71490 |
| 71691 | NW_011499910.1 | exon | LOC105133891 | protein CHROMATIN REMODELING 20-like |
| 83206 | NW_011499925.1 | gene | LOC105135597 | probable 28S rRNA (cytosine(4447)-C(5))-methyltransferase |
| 88648 | NW_011500732.1 | gene | LOC105116181 | G-protein coupled receptor 1 |
| 95857 | NW_011499944.1 | exon | LOC105137304 | ABSCISIC ACID-INSENSITIVE 5-like protein 2 |
| 97793 | NW_011499947.1 | gene | LOC105137665 | ATP-dependent DNA helicase DDM1-like |
| 102860 | NW_011499954.1 | gene | LOC105138234 | trafficking protein particle complex subunit 8 |
| 117080 | NW_011499977.1 | gene | LOC105139603 | putative cyclic nucleotide-gated ion channel 15 |
| 125456 | NW_011499992.1 | gene | LOC105140440 | persulfide dioxygenase ETHE1 homolog |
| 132164 | NW_011500803.1 | exon | LOC105116423 | nuclear transcription factor Y subunit A-7-like |
| 137999 | NW_011500014.1 | exon | LOC105141593 | probable WRKY transcription factor 72 |
| 146864 | NW_011500029.1 | exon | LOC105142390 | G-type lectin S-receptor-like serine/threonine-protein kinase At4g27290 |
| 148974 | NW_011500034.1 | gene | LOC105142610 | proteasome subunit alpha type-5 |
| 153370 | NW_011500042.1 | gene | LOC105107299 | thioredoxin-like 1-1%2C chloroplastic |
| 155187 | NW_011500445.1 | gene | LOC105114738 | far upstream element-binding protein 2-like |
| 163163 | NW_011500460.1 | exon | LOC105114822 | probable glycosyltransferase At3g07620 |
| 164421 | NW_011500063.1 | gene | LOC105108094 | protein SENSITIVITY TO RED LIGHT REDUCED 1 |
| 168577 | NW_011500072.1 | gene | LOC105108522 | lysine-specific demethylase JMJ25-like |
| 173403 | NW_011500084.1 | gene | LOC105108913 | cationic amino acid transporter 2, vacuolar-like |
| 174370 | NW_011500086.1 | gene | LOC105108966 | mannose/glucose-specific lectin-like |
| 181824 | NW_011500103.1 | exon | LOC105109520 | TMV resistance protein N-like |
| 188510 | NW_011500117.1 | gene | LOC105109972 | tRNA:m(4)X modification enzyme TRM13 homolog |
| 190540 | NW_011500121.1 | gene | LOC105110122 | serine/threonine-protein kinase tricorner-like |
| 192994 | NW_011500927.1 | exon | LOC105116838 | serine/arginine repetitive matrix protein 5-like |
| 196423 | NW_011501335.1 | exon | LOC105117711 | disease resistance protein RPM1-like |
| 206716 | NW_011500161.1 | gene | LOC105111188 | microfibrillar-associated protein 1-like |
| 213286 | NW_011500176.1 | gene | LOC105111440 | probable histone-arginine methyltransferase 1.4 |
| 214368 | NW_011500179.1 | gene | LOC105111482 | putative CCA tRNA nucleotidyltransferase 2 |
| 215570 | NW_011500182.1 | gene | LOC105111539 | serine carboxypeptidase-like 51 |
| 221123 | NW_011500194.1 | gene | LOC105111754 | dol-P-Man:Man(6)GlcNAc(2)-PP-Dol alpha-1, 2C2-mannosyltransferase-like |
| 234755 | NW_011500224.1 | exon | LOC105112339 | auxin response factor 17-like |
| 237213 | NW_011500232.1 | exon | LOC105112451 | mitogen-activated protein kinase 2-like |
| 239255 | NW_011500238.1 | exon | LOC105112539 | putative G3BP-like protein%2C transcript variant X3 |
| 241633 | NW_011501042.1 | exon | LOC105117132 | serine/threonine-protein kinase CDL1-like |
