# Supplementary material for: Heterophylly Quantitative Trait Loci Respond to Salt Stress in the Desert Tree Populus euphratica
Source: Front Plant Sci. 2021 Jul 15;12:692494. doi: 10.3389/fpls.2021.692494 (PMC8321784; doi:10.3389/fpls.2021.692494)
Supplement: Supplementary file 1 [file Data_Sheet_1.zip › Table S2.DOCX]

**Table S2**. Gene information of significant SNPs for salt condition.

| Number | Chromosome ID | Distribution | Gene ID | Annotation |
| --- | --- | --- | --- | --- |
| 3353 | NW_011499846.1 | gene | LOC105131169 | glycine--tRNA ligase 2%2C chloroplastic/mitochondrial |
| 3594 | NW_011499846.1 | exon | LOC105131868 | F-box protein At5g51370-like |
| 6878 | NW_011499848.1 | gene | LOC105112394 | serrate RNA effector molecule |
| 8441 | NW_011499849.1 | gene | LOC105120488 | BEACH domain-containing protein lvsC |
| 10706 | NW_011499850.1 | gene | LOC105121124 | adenylate kinase 5, 2C chloroplastic |
| 18435 | NW_011499856.1 | exon | LOC105123276 | auxin transport protein BIG |
| 18817 | NW_011499856.1 | exon | LOC105123321 | proline dehydrogenase 2%2C mitochondrial-like |
| 24120 | NW_011499860.1 | exon | LOC105124579 | 50S ribosomal protein L17%2C chloroplastic%2C transcript variant X2 |
| 24500 | NW_011499860.1 | gene | LOC105124653 | guanosine nucleotide diphosphate dissociation inhibitor At5g09550 |
| 25919 | NW_011499862.1 | gene | LOC105125089 | probable disease resistance protein At4g27220 |
| 31094 | NW_011499866.1 | gene | LOC105126240 | protein LHY-like |
| 31663 | NW_011499867.1 | gene | LOC105126350 | RNA polymerase sigma factor sigD%2C chloroplastic |
| 33097 | NW_011499868.1 | exon | LOC105126665 | probable leucine-rich repeat receptor-like protein kinase At1g35710 |
| 37026 | NW_011499873.1 | gene | LOC105127854 | oxysterol-binding protein-related protein 1C |
| 39224 | NW_011499875.1 | gene | LOC105128328 | tobamovirus multiplication protein 2A-like |
| 46809 | NW_011499883.1 | gene | LOC105129804 | adenylyl-sulfate kinase 3-like |
| 46844 | NW_011499883.1 | exon | LOC105129876 | mitochondrial outer membrane protein porin 2-like |
| 48054 | NW_011499884.1 | gene | LOC105130009 | protein argonaute 10 |
| 48552 | NW_011499885.1 | gene | LOC105130122 | translation initiation factor IF-2, 2C chloroplastic-like |
| 63006 | NW_011499900.1 | exon | LOC105132686 | ribulose bisphosphate carboxylase/oxygenase activase, 2C chloroplastic |
| 65447 | NW_011499902.1 | gene | LOC105133021 | phenolic glucoside malonyltransferase 1-like |
| 70630 | NW_011499909.1 | exon | LOC105133783 | mediator-associated protein 2 |
| 74624 | NW_011499914.1 | exon | LOC105134415 | protein ENHANCED DISEASE RESISTANCE 2-like |
| 74652 | NW_011499914.1 | gene | LOC105134419 | F-box protein SKIP16-like |
| 76915 | NW_011499917.1 | exon | LOC105134746 | phospholipase A-2-activating protein-like |
| 87980 | NW_011499931.1 | gene | LOC105136047 | protein RRP5 homolog%2C transcript variant X1 |
| 91599 | NW_011499936.1 | gene | LOC105136522 | probable LRR receptor-like serine/threonine-protein kinase At1g53440 |
| 92229 | NW_011499937.1 | gene | LOC105136635 | protein ROOT HAIR DEFECTIVE 3 homolog 2-like |
| 92321 | NW_011499937.1 | exon | LOC105136652 | exocyst complex component EXO84C-like |
| 100881 | NW_011499951.1 | gene | LOC105138001 | nodal modulator 1 |
| 107273 | NW_011499960.1 | gene | LOC105138604 | inositol-tetrakisphosphate 1-kinase 1-like |
| 117585 | NW_011499978.1 | exon | LOC105139638 | probable fructokinase-5 |
| 122120 | NW_011499985.1 | gene | LOC105140060 | 3-hydroxyisobutyryl-CoA hydrolase 1-like |
| 135545 | NW_011500009.1 | exon | LOC105141358 | diaminopimelate epimerase, 2C chloroplastic-like |
| 137739 | NW_011500413.1 | gene | LOC105114517 | thiamine biosynthetic bifunctional enzyme TH1%2C chloroplastic |
| 147404 | NW_011500030.1 | gene | LOC105142408 | glutamyl-tRNA(Gln) amidotransferase subunit A |
| 147414 | NW_011500030.1 | exon | LOC105142410 | cell division cycle protein 27 homolog B |
| 154985 | NW_011500045.1 | gene | LOC105107433 | E3 ubiquitin-protein ligase SHPRH-like |
| 155622 | NW_011500046.1 | gene | LOC105107495 | probable LRR receptor-like serine/threonine-protein kinase At1g07650 |
| 164961 | NW_011500064.1 | gene | LOC105108152 | uncharacterized membrane protein At1g16860-like |
| 181805 | NW_011500103.1 | exon | LOC105109520 | TMV resistance protein N-like |
| 184183 | NW_011500907.1 | gene | LOC105116788 | ABC transporter C family member 2-like |
| 193592 | NW_011500129.1 | gene | LOC105110346 | putative respiratory burst oxidase homolog protein H |
| 195571 | NW_011500134.1 | gene | LOC105110445 | protein MON2 homolog |
| 195578 | NW_011500134.1 | gene | LOC105110446 | pre-rRNA-processing protein TSR2 homolog |
| 195642 | NW_011500134.1 | exon | LOC105110458 | WD repeat-containing protein 48-like |
| 203590 | NW_011500153.1 | exon | LOC105111037 | NADP-dependent alkenal double bond reductase P2-like |
| 207263 | NW_011500162.1 | gene | LOC105111194 | ABC transporter F family member 1-like |
| 208788 | NW_011500165.1 | exon | LOC105111238 | TMV resistance protein N-like |
| 209511 | NW_011500167.1 | exon | LOC105111272 | lysine-specific demethylase JMJ18-like |
| 212215 | NW_011500573.1 | gene | LOC105115456 | inactive protein kinase SELMODRAFT_444075-like |
| 212825 | NW_011500175.1 | gene | LOC105111425 | ARF guanine-nucleotide exchange factor GNOM-like |
| 217774 | NW_011500186.1 | gene | LOC105111615 | histone deacetylase 9 |
| 224181 | NW_011500199.1 | gene | LOC105111847 | rhodanese-like domain-containing protein 11%2C chloroplastic |
| 228229 | NW_011500208.1 | gene | LOC105112064 | pentatricopeptide repeat-containing protein At1g62930, 2C chloroplastic-like |
| 237066 | NW_011500231.1 | gene | LOC105112447 | bifunctional epoxide hydrolase 2-like |
| 238310 | NW_011501034.1 | exon | LOC105117107 | chromo domain-containing protein LHP1-like |
